# *BmMed6* modulates mating behavior by ORs and antennae structural genes in the silkworm

**DOI:** 10.1016/j.isci.2025.112017

**Published:** 2025-02-13

**Authors:** Zhang Liying, Yang Dehong, Tang Longhao, Wei Xiangyi, Li Kai, Huang Yongping

**Affiliations:** 1 College of Life Science, East China Normal University, Shanghai 200062, China; 2 School of Environmental Science and Engineering, Shanghai Jiao Tong University, Shanghai 200240, China; 3 College of Chemistry and Chemical Engineering, Shanghai University of Engineering Science, Shanghai 201620, China

**Keywords:** Biological sciences, Entomology, Molecular biology

## Abstract

Gene expression is under strict and precise control to regulate organism development and maintain various physiological functions. The Mediator complex is a regulator of gene transcription. Our study focused on *BmMed6*, a component of the Mediator complex in the *Bombyx mori*. We construct *BmMed6* mutants using the CRISPR-Cas9 system. The mutants exhibited abnormal growth patterns in their antennae, which limited their mating behavior. RNA-seq and gene expression analysis have revealed that the expression of genes associated with structural constituents of the cuticle in the antennae of the mutant was aberrant. Moreover, the deficiency of *BmMed6* also caused the downregulation of olfactory receptor genes. Our findings offer novel insights into the biological role of *BmMed6* in antenna growth, revealing its crucial role in regulating antenna structure and olfactory gene expression to influence mating behaviors. This discovery identifies *BmMed6* as a viable new target gene for pest control.

## Highlights

• *BmMed6* is required for the development of antenna in the silkworm
• *BmMed6* regulate the ORs and antennae structural genes
• The abnormal antennae influence mating behaviors

## Graphical abstract

**Figure:**
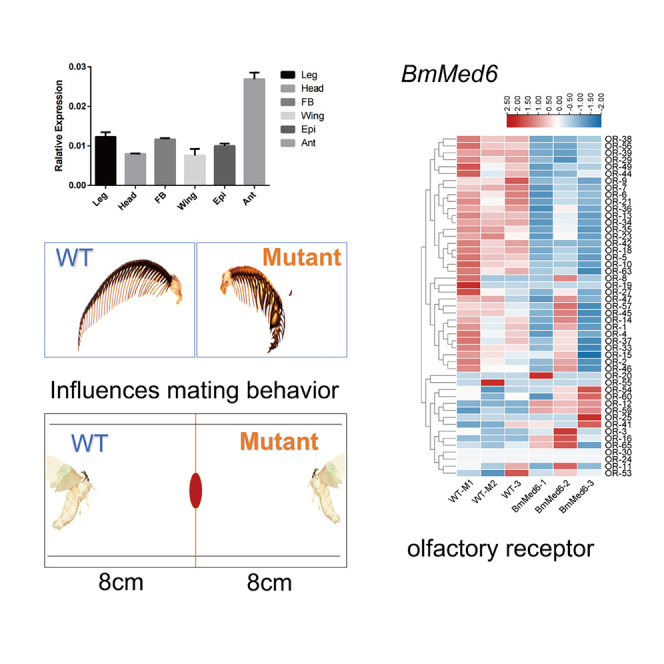

## Introduction

The transcription of cells is a complex biological process. This fundamental, conserved, and highly intricate biological process is tightly regulated to ensure that the genetic program adapts to the cellular demands. Dysregulation of transcription can lead to serious growth and physiological function disorders.^1^ In eukaryotic cells, transcriptional machinery and specialized factors regulate RNA synthesis.^2^ RNA polymerase II (Pol II) enzyme transcribes all protein-coding genes, and is globally regulated by the Mediator.^3^

Mediator is a large conformationally flexible protein complex consisting of variable subunits required for transcription activation in all eukaryotes.^4^ The Mediator complex regulates gene expression and is a general transcription factor.^5^^,^^6^ It is essential for the transcription of nearly all genes transcribed by RNA polymerase B.^4^^,^^7^^,^^8^ Mediator subunits are the main targets of DNA-binding transcription factors (TF) activation domains.^9^ Mediator communicates regulatory signals from DNA-bound TF directly to the RNA polymerase II (Pol II) enzyme.^10^^,^^11^^,^^12^^,^^13^ So, Mediator is widely involved in the information transfer, which is called the central controller of gene transcription in eukaryotes.^3^

The function of the Mediator complex is evolutionarily conserved despite changes in its subunit composition, sequences, and overall structure over evolutionary time.^14^ Notably, the subunits of the Mediator complex exhibit variability instead of being strictly composed of single isoforms.^14^ This diversity underscores the complexity and adaptability of the Mediator complex in gene transcription across diverse organisms.^15^ Structurally, the Mediator complex consists of several modules, including central module-terms head, middle, tail, and CDK8 kinase modules (CKMs).^16^^,^^17^ Each module comprises several subunits. It appears that the majority of these subunits perform specific functions.^3^^,^^18^^,^^19^ In mammals, the Mediator complex comprises up to 30 subunits, while in yeast, there are 25 subunits.^1^ Different TFs bind to specific Mediator subunits, indicating the complex’s ability to mediate diverse transcriptional responses. Studies in yeast have demonstrated that the Mediator complex affects almost all protein expression regulation, and knocking out any one of the mediator subunits, including Med4, Med6, Med7, Med8, Med10, Med11, Med14, Med17, Med21, and Med22, results in lethality.^7^^,^^20^^,^^21^ This underscores the critical role of the Mediator complex in transcriptional regulation. In *Drosophila*, the Mediator complex is implicated in innate immunity^22^ and has an influence on developmental processes.^23^^,^^24^ Additionally, CDK8 and CycC, together with a few other subunits of the Mediator complex may collaborate with other transcriptional cofactors to modulate Mad-dependent transcription during the development of wings.^25^ That further provides evidence of its widespread and essential functions. It has been proposed that the specificity of Mediator subunits comes from their ability to interact with specific transcription factors, thereby mediating their regulatory activity.^9^^,^^26^ In *Drosophila*, *intersex* serves as a subunit of the Mediator complex, collaborating with the products of *dsx* to regulate sex determination and differentiation.^27^^,^^28^ It is predicted that there exist an interaction between *ix* and *Med6*.^29^ In our previous studies, we found that *intersex* functions in the normal development of female external genitalia and regulates imaginal disc morphogenesis in the silkworm.^30^

*Med6* is the main component of large conformationally flexible Mediator protein complexes, but the role of *Med6* in Mediator function remains uncomplicated. Studies on *Saccharomyces cerevisiae* had found that *Med6* was required for cell viability, proliferation and activated transcription.^31^^,^^32^ Furthermore, RNA interferes with the *CeMed6*, *CeMed7*, and *CeMed10*/*CeNut2* genes in *C.elegans*, demonstrates the essentiality of the Mediator complex in regulating developmental and regulatory genes in *Caenorhabditis elegans*.^33^^,^^34^ Among these genes, several are involved in cellular proliferation and metabolism. This indicated the critical role of *Med6* in the developmental processes and cell transcription.^35^ In *Drosophila*, *Med6* mutants die at the third-instar larvae stage or experience unsuccessful transition to pupate due to severe proliferation defects in mitotic cells. The transcriptional regulation of numerous genes is impacted.^35^ However, limitations arise from the inability of mutant larvae to progress beyond the third instar stage, thus hindering our understanding of later developmental stages. This highlights the need for further research to comprehend the broader implications. These findings all indicate the pivotal role of *Med6* in the recruitment function of the Mediator complex, which is critical for Mediator’s regulation of gene transcription and plays a significant role in growth and development. However, for a comprehensive understanding, it is imperative to consider alternative model organisms, such as the silkworm (*Bombyx mori*).

In this study, we explored the function of *BmMed6* in silkworms using CRISPR/Cas9 system. The *BmMed6* mutants successfully underwent pupation and eclosion compared to *Drosophila*.^35^ The importance of complementary studies in diverse species for a more comprehensive understanding of the intricate regulatory networks governing developmental processes and cell transcription. The deficiency of *BmMed6* significantly influenced the growth and development of silkworms, resulting in malformed antennae and impaired mating behavior. Specifically, *BmMed6* was found to influence the expression of genes responsible for the structural constituent of the cuticle, sensory perception of smell and olfactory receptor activity. In our study, the successful pupation in the mutants provides unique advantages for understanding the function of *BmMed6* at pupal and adult stages in the silkworm. Consequently, this discovery highlights *BmMed6* as a promising novel target gene for precision lepidopteran pest control strategies.

## Results

### Constructing the *BmMed6* mutants by a transgenic binary CRISPR/cas9 system

**Figure 1:**
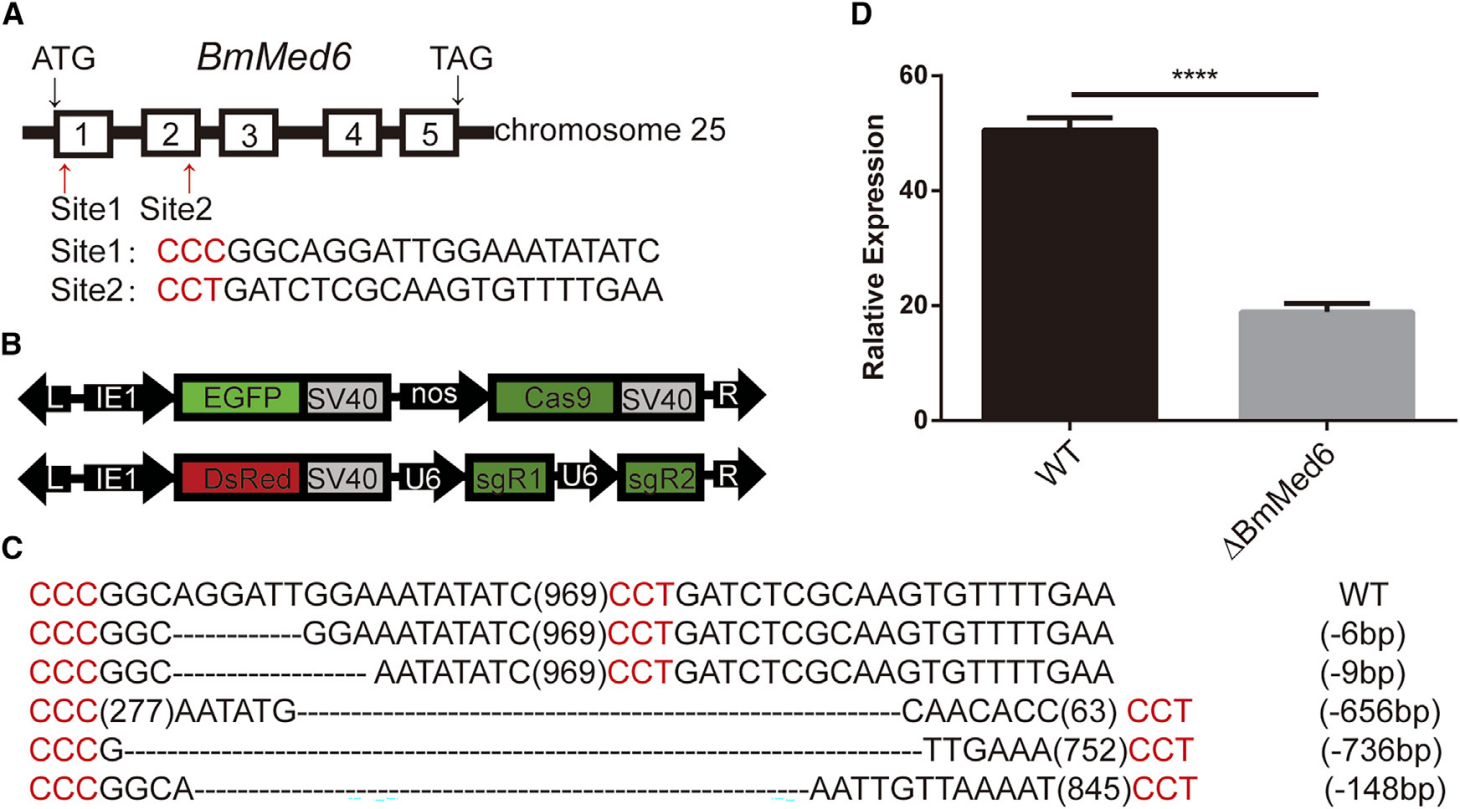
Successfully constructed the *BmMed6* mutant by a binary CRISPR/cas9 system (A)The gene structure and coding exons of *BmMed6*. The box represents the five coding exons. The red arrows indicate the two sites targeted by sgRNA. (B) Schematic of the binary transgenic vectors. (C) The genotype of the WT and mutants. The top line shows the WT genotype, and the sequence of the mutants is displayed at the down lines. The sequence of mutations in mutant individuals which produced by crossing nos-Cas9 and U6-BmMed6 sgRNA transgenic silkworm lines. The PAM sequence is shown in red, and the number of the nucleotides deleted is indicated on the right. (D) The mRNA level of the WT and mutant. The asterisks indicate the significant differences compared to the WT. Data are presented as mean ± SEM. Statistically significant differences were determined by paired Student’s t-test. ∗∗∗∗, *p* < 0.0001.

We compared the Med6 protein sequences (Figure S1) across multiple species, indicating that Med6 is relatively conserved evolutionarily (Figure S1). To study the function of Mediator in silkworm development, we used a binary transgenic CRISPR/Cas9 system, which has been described in the previous study, to construct the *BmMed6* mutants.^36^^,^^37^^,^^38^ The *BmMed6* gene has 5 exons on chromosome 25. We designed two sgRNAs that target exon 1 and exon 2 (Figure 1A). We used nos-Cas9 transgenic strain to express Cas9, *EGFP* as a screening marker. U6-sgRNA transgenic strain expresses two site-specific driven by U6 promoter, *dsRed* red fluorescence as a marker (Figure 1B). The F1 generation was obtained by crossing two transgenic lines. The individuals expressing both Cas9 and sgRNAs were *BmMed6* mutants. To detect mutations, the DNA lesions were confirmed by random detection of F1 progeny by PCR (Figure 1C). Through qPCR validation, the mRNA level also exhibited a significant decrease (Figure 1D). The results showed that we got the *BmMed6* mutant successfully.

### *BmMed6* deficiency induced reduction in body size of mutant individuals

In yeast and *Drosophila*, *Med6* is crucial for transcriptional regulation of many developmental genes, but insights from *dMed6* mutants are limited to early stages^31^^,^^32^^,^^35^; thus, exploring alternative models like silkworms is imperative for deeper understanding.

**Figure 2:**
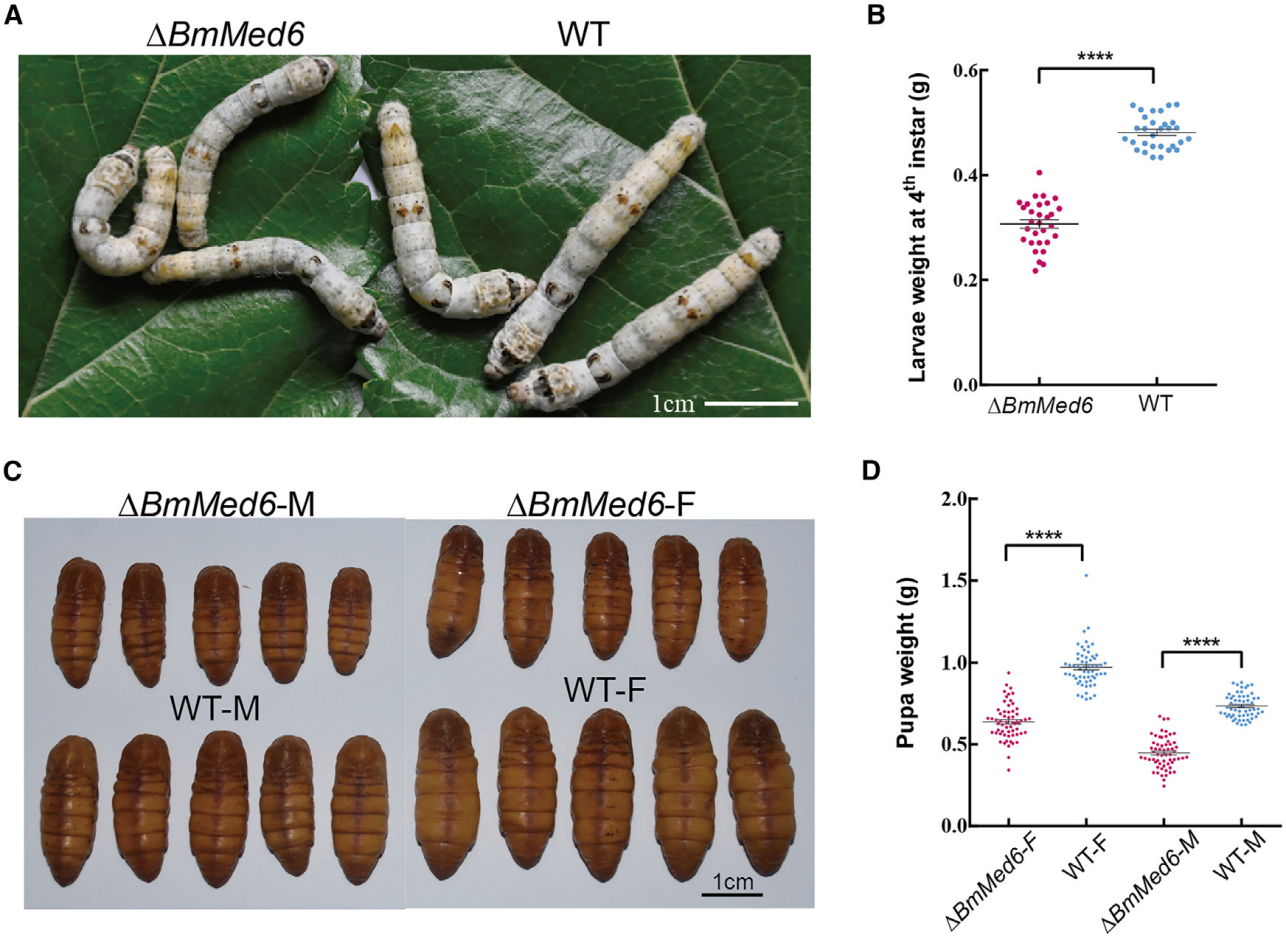
Mutant larvae and pupae are smaller than wild-type (A) Image of the larvae. (B) Larval weight statistics show a decrease in weight. (C) Image of the pupae. Both female and male mutant pupae are smaller than those of wild types. (D) Pupal weight statistics reveal that mutant pupae are lighter than wild-type pupae. Scale bars are positioned at the lower-right corner in white or black. Data are presented as mean ± SEM. Statistically significant differences were determined by paired Student’s t-test. ∗∗∗∗, *p* < 0.0001.

In silkworms, the mutant individuals exhibited a decrease in size and weight throughout their developmental stages (Figure 2). During the larval stage, mutants showed a reduction in individual size and weight (Figures 2A and 2B). This trend persisted through the pupal stage (Figure 2C and 2D), suggesting that the knockout of the *BmMed6* gene had a profound impact on the normal development of silkworms, *BmMed6* plays a crucial role in the regulation of growth and developmental processes. Despite these developmental abnormalities, the mutants were able into the adult stage following the pupal stage, allowing for the investigation of the biological functions of the *BmMed6* during both pupal and adult stage. Those phenotypes are not only an issue of pupal size and weight reduction but also indicate potential dysregulation of the complex biological processes underlying insect morphology.

### Loss function of *BmMed6* caused defective antennal structures

**Figure 3:**
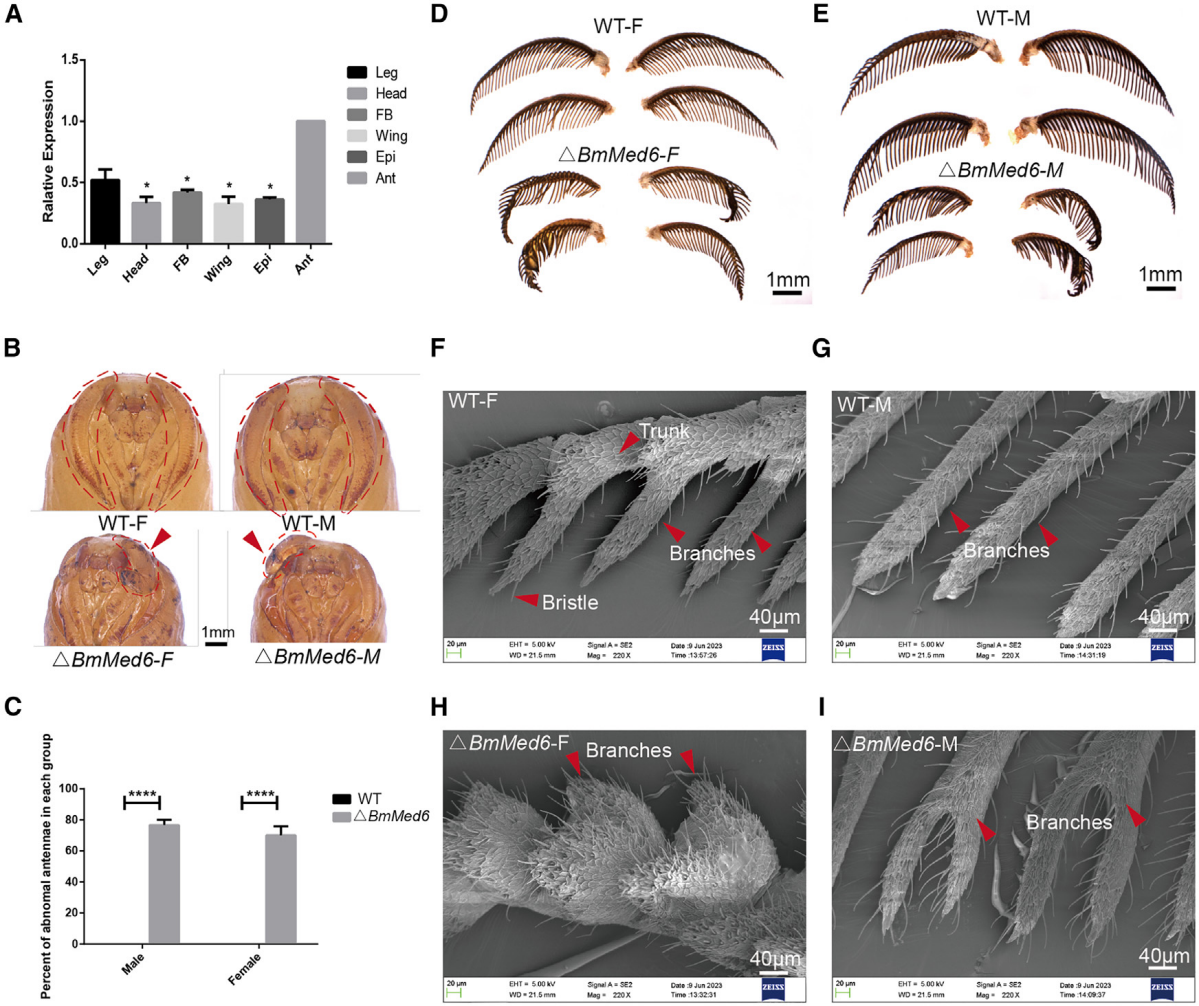
Loss of *BmMed6* impairs antennal structures (A) The relative mRNA levels of *BmMed6* in leg, head, fatbody (FB), wing, epidermis (Epi) and antenna (Ant) were determined by qRT-PCR. (B) The head and antennae of the pupal stage. The red dotted line showed outlines and segmentation of the antenna. The red arrows indicate the anomalous antennae (C) Rate of malformation of antennae. (D) The antennae of female silk moths. (E) The antennae of male silk moths. (F–I) Observation of the antennae by SEM. Scale bars are positioned at the lower-right corner in white or black. Data are presented as mean ± SEM. Statistically significant differences were determined by paired Student’s t-test. ∗, *p* < 0.05, ∗∗∗∗, *p* < 0.0001.

In *Drosophila*, *dMed6* mRNAs increased during the early stages of pupation and reached its highest level at adulthood.^35^ Here, we detected the mRNA levels of *BmMed6* in the leg, head, fat body, wing, cuticle and antenna in the silk moth by qRT-PCR at adult stage. It showed that *BmMed6* exhibited the highest mRNA expression levels in the antennae of silk moth, suggesting its potential role in antenna development (Figure 3A).

During the pupal stage, there are antennal pockets on the ventral surface in the wildtype (WT). The prototype of the antennae was one-layered, leaf-shaped epidermal (Figure 3B, top line). Interestingly, we found that *BmMed6* mutations exhibited abnormal antennal structures. From the pupal stage, abnormal antennae outline and segmentation have been observed in the mutants (Figure 3B). The cuticle of the large antennal pockets exhibited an unusual curl segmentation pattern of the antenna. The antennae become smaller and deformed (Figures 3D and 3E), with a malformation rate of 76% in males and 70% in females (Figure 3C).

Adult antennae and neurons develop after the onset of pupation. The silk moth bipectinate antenna has a main trunk and two rows of comb-like branches.^39^ In the mutants, SEM pictures revealed aberrant structural characteristics of the antennae, which are characterized by shortened, underdeveloped, and atypical branching patterns (Figures 3F–3I). On the surface of the cuticular specialization on the antennae, there are thousands of olfactory sensilla (olfactory receptor neurons) with high efficiency of capturing odor molecules.^40^ The capability of male moths to locate their mates from afar, facilitated by the utilization of airborne pheromone signals.^41^ Structural deformities of the antennae may cause physiological and behavioral abnormalities. These phenotypes of mutants all suggest that *BmMed6* may play a role in antennae development and function.

### Disordered expression of genes linked to antennae structural deformities in the *BmMed6* mutant

**Figure 4:**
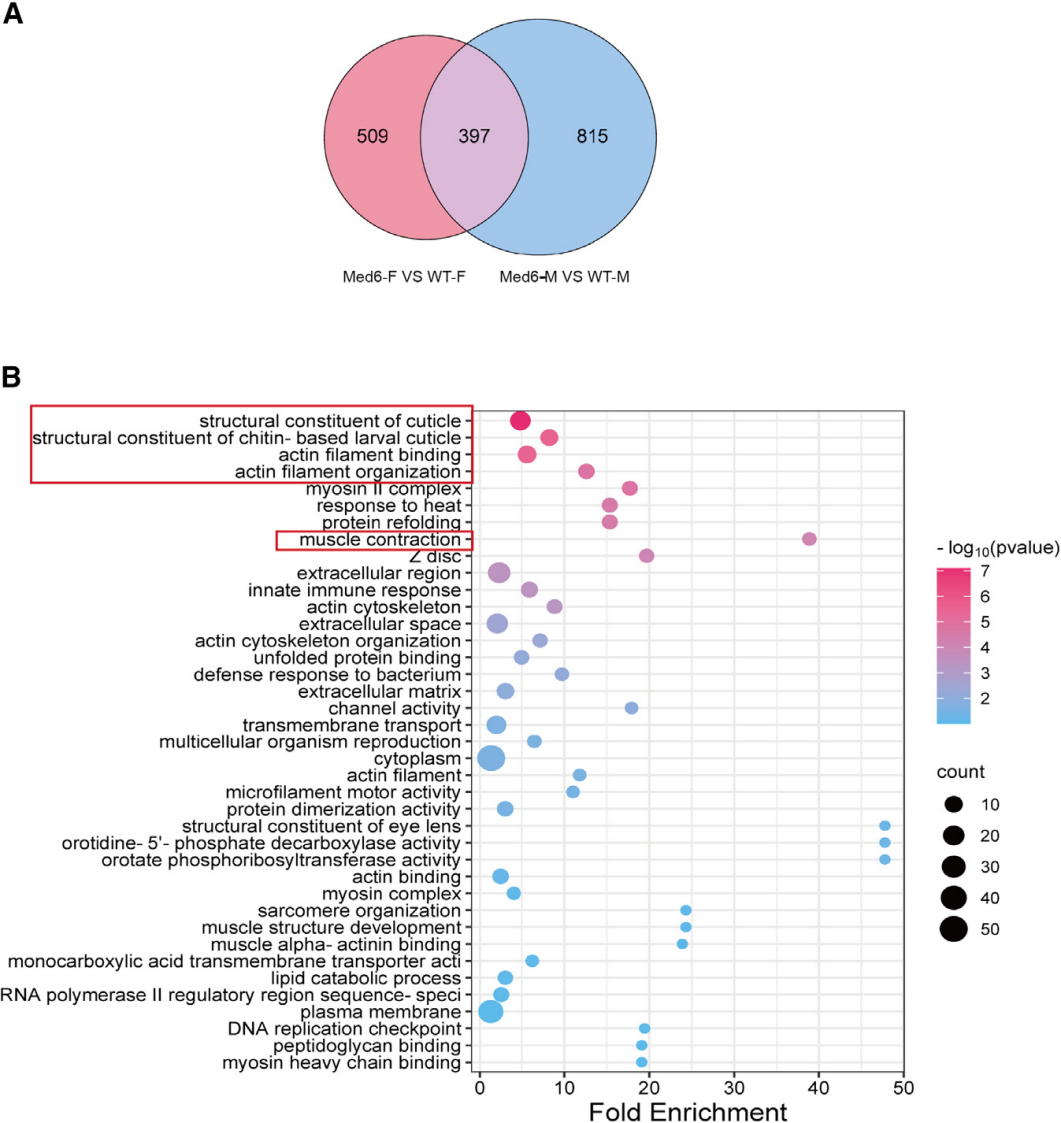
RNA-seq data analysis reveals disordered expression of genes linked to antennae structural deformities in the *BmMed6* mutant (A) Venn diagram depicting differentially expressed genes. (B) The functional categories of commonly differentially expressed genes between females and males in the GO database.

Given the antennal deformities observed in the mutant, we aimed to assess its impact on genes related to antennal structure and other associated factors. To investigate genes involved in antennae development regulated by *Med6*, we performed RNA-seq of antennae. A total of 16711 genes were identified, among which 906 genes exhibited differential expression genes (DEGs) in female antennae compared to the WT of these, 349 genes were downregulated, while 557 genes were upregulated (Figure S2). In male antennae, we identified 1212 differentially expressed genes, including 622 downregulated and 590 upregulated, compared with the WT (Figure S2). The GO analysis categorizes and annotates the differentially expressed genes obtained from transcriptome sequencing, including behavior, developmental process, receptor regulator process, which are displayed in the supplementary figures. Additionally, KEGG enrichment analysis has been performed to reveal the sensory system and cell growth pathways involved (Figure S5). Since the antenna structures of both females and males have changed, we focused on the 395 genes that were differentially expressed in both male and female mutants (Figure 4A). The Gene Ontology (GO) terms enriched in structural constituent of cuticle, structural constituent of chitin-base larval cuticle, actin filament binding, actin filament organization, muscle contraction (Figure 4B). Furthermore, a heatmap analysis was performed, revealing an upregulation in the mRNA expression levels of these genes (Figure S2B). These findings suggest that genetic alterations related to structure may underlie the observed antennal deformities.

### The defective antennae affected the mating behavior in the *BmMed6* male mutants

**Figure 5:**
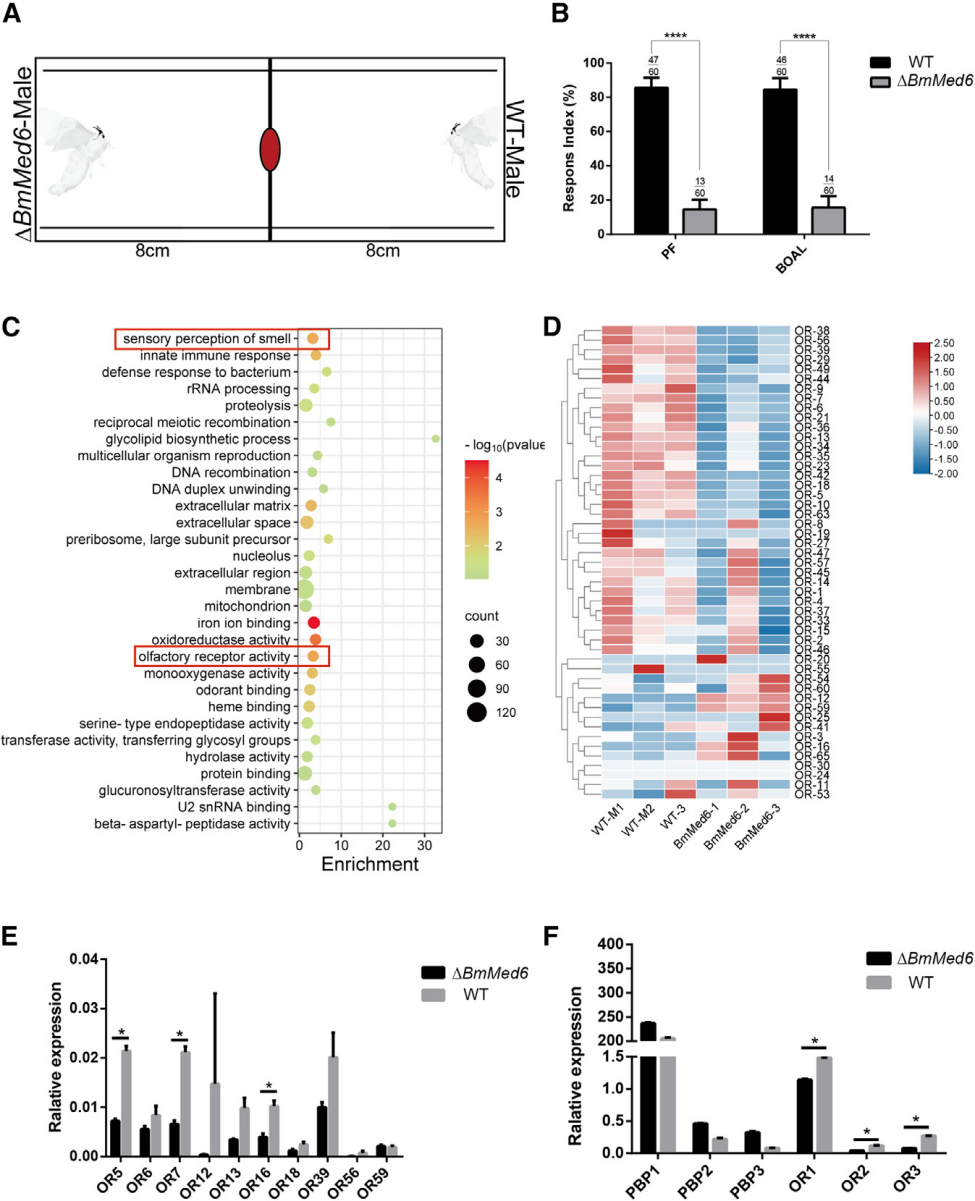
Analysis of mating behavior in adult silkworm (A) Schematic representation of mating behavior assay. The moths on both sides represent WT and mutant male moths, and the red circle in the middle represents the female moths or filter paper with pheromone components. (B) Coupled bar plots of competitive mating behavior between WT and mutant silkworms. Data are represented as mean ± SEM. (C) The functional categories of the significantly downregulated differentially expressed genes of males were identified in males in the Gene Ontology (GO) database. (D) Heatmap of OR genes from WT and mutant. (E and F) mRNA levels of OR genes in the antennae of WT and mutant moth. Data are presented as mean ± SEM. Statistical significance was determined by Student’s *t* test, ∗, *p* < 0.05, ∗∗∗∗, *p* < 0.0001, n. s. not significant (*p* > 0.05). PF, natural pheromone released by a wild-type female moth, BOAL, the mixture of bombykol and bombykal in a ratio of 11:1.

During the mating process of silkworms, the sex pheromone secreted by the female silkworm attracts male moths. After the olfactory receptors on the antennae of male moths receive this chemical signal, they will become extremely excited for mating.^42^ The male antennae play a key role in the recognition of sex pheromone, which is important for successful mating. Could the deformation observed in the antennae of the mutant individuals potentially lead to alterations in certain physiological behaviors? To elucidate the role of *BmMed6* in silk moth mating behavior, we executed a competitive choice assay in a plastic enclosure. A female moth or bombykol/bombykal (sex pheromone component) was placed at the center,^43^ with one *BmMed6* mutant male silkmoth and one WT male silkmoth positioned 8 cm away on either side (Figure 5A). The male moth reached the midline first and counted it as one point. Statistical analysis revealed that the mutant males always reached the midline more slowly than the WT males. The wild-type males were more willing to detect female moths or pheromones and engage in mating (Figure 5B). The mutant displayed weak ability to perceive pheromones, suggesting that *BmMed6* plays a role in antennae perception and subsequently mating behavior.

### *BmMed6* mutant cause OR dysregulation

The Mediator complex is the bridge between transcription factors and machinery, orchestrating the occurrence of gene transcription, is crucial for transcriptional regulation.^11^ To investigate whether the mutant’s impairment in pheromone perception is due to malformed antennae or a direct regulation of genes involved in pheromone sensing, we focused on its downregulated genes in the *BmMed6* mutant. Through transcriptome analysis of significantly downregulated genes, the Gene Ontology (GO) terms enriched in “sensory perception of smell” and “olfactory receptor activity” (Figure 5C). Numerous olfactory sensilla and their associated neurons in the silk moth antennae are responsible for trapping pheromone.^44^ The male moths primarily rely on the olfactory receptors on their antennae to receive such signal. Given that pheromone perception is accomplished by OR receptors.^45^ We investigate potential alterations in OR mRNA levels. A heatmap for the OR gene transcription of WT and *BmMed6* mutant showed obvious different transcript patterns for OR genes (Figure 5D). The results of qPCR verification experiments were consistent with the significantly differentially expressed OR gene (Figure 5E). The results indicated that the deletion of *BmMed6* could cause OR down-regulation, which in turn regulated pheromone perception. The perception ability of male moths to pheromones has declined, which is not only due to structural abnormalities, but also to the changes in the genes like OR that perceive pheromones.

Male silk moths detect female pheromones by antennae, which elicits mating behavior.^44^^,^^46^^,^^47^ *BmOR1* and *BmOR3*, sex odorant receptors, exhibit male-specific expression and exhibit specificity for bombykol and bombykal, respectively.^45^ *BmPBP1*, an insect pheromone-binding protein, also shows male-biased expression and is essential for the selective binding of bombykol by *BmOR1*.^48^^,^^49^ Here, we test the expression of genes *BmOR1* and *BmOR3*, *BmPBP1*, *BmPBP*2, and *BmPBP3*. The expression levels of mRNA downregulated in *OR1*, *OR2*, and *OR3*, as well as the unchanged in *PBP1*, *PBP2*, and *PBP3* (Figure 5F). Many olfactory receptors in the silk moth antennae are responsible for detecting pheromones.^50^^,^^51^ Therefore, we further investigated the mutant moths’ perception of pheromones, revealing a decreased ability in male moths to detect these chemical signals (Figure 5B). The results suggest that the loss of *BmMed6* results in downregulation of ORs, modulating pheromone perception, thereby highlighting the influence of *BmMed6* deficiency on male-specific pheromone receptors.

## Discussion

We evaluated the function of the *Med6* gene in the Lepidopteran model insect *B. mori* by employing CRISPR/Cas9-mediated deletion of the *BmMed6* genome sequence. The mutant displayed abnormal antenna morphology and growth restriction. Specifically, the antennae undergo a reduction in size and distortion, exhibiting shortened, underdeveloped, and atypical branching structures. Subsequent RNA-seq analyses identified differential gene expression in the structural constituent of cuticle, sensory perception of smell, and olfactory receptor activity pathway, particularly implicating *BmMed6* in modulating genes associated with antennal morphology. These molecular changes likely contribute to the observed deformations. The defective antennae in the *BmMed6* mutants noticeably impacted mating behavior, wherein moths exhibited reluctance to mate. This behavior indicates that the deformities in antennae restricted mating.

The adult antenna initiates its development during pupation, with numerous adult neurons arising within the simple epidermis and aligning toward the thicker exterior of the newly formed pupal cuticle.^39^ The main components of insect cuticle are chitin and protein.^52^ Changes in the pathways related to the structural constituents of cuticle, structural constituents of chitin-based larval cuticle in the antennae of *B. mori* can affect the formation of antennae (Figure 4B). There are numerous olfactory sensilla and their associated neurons in the silk moth antennae, which are primarily responsible for pheromone detection.^44^^,^^53^ Consequently, the observed deformation in the antennae (Figures 3D and 3E) may lead to alterations in certain physiological behaviors, given that pheromone perception heavily relies on the expression of OR genes in the antennae. Silk moth uses its wings to direct pheromones from its anterior, allowing another silk moth to receive and perceive the signal from the source of the pheromones via its antennae.^54^^,^^55^ This assertion is supported by the observed downregulated in the expression of OR genes in the antennae of mutant individuals (Figure 5D). Therefore, compromised pheromone reception due to antennal alterations ultimately resulted in restricted mating behavior (Figure 5B), highlighting the crucial role of *BmMed6* in regulating antennal morphology and, consequently, mating.

The previous study verified that *Saccharomyces cerevisiae Med6* is required for cell viability and proliferation,^31^ and *Drosophila Med6* is required for elevated expression of a large but distinct set of developmentally regulated genes.^35^ While valuable insights have been gained from investigations into *dMed6* mutants in *Drosophila*, it is essential to recognize the limitations of this model system. These limitations notably encompass the incapacity of *dMed6* mutants to advance beyond the third larval instar stage, thereby limiting our understanding of subsequent developmental phases. Here, in Lepidopteran *Bombyx mori*, mutants of *BmMed6* have been able to successfully pupate and emerge as adults. The role of *Med6* during the pupal and adult stages has been investigated in the silkworm. Particularly, in terms of affecting antennal development *Med6* regulates the expression of genes related to antennal structure, as well as downregulates the expression of the OR gene. This leads to abnormalities in the antennal structure and impedes pheromone reception, thereby severely impacting mating behavior.

*BmMed6* affects growth and development, but we only observed this phenomenon in this study and did not delve into it. As for the effect of *BmMed6* on antennal development, we showed transcriptome analysis that the results of *BmMed6* mutant and wild-type antennae structure. There were differences in structural constituent of cuticle, sensory perception of smell, and olfactory receptor activity pathway, and the expression of OR genes was disordered. Those proteins in structural constituent of cuticle pathways are components that constitute the antennae.^56^ However, it remains unclear whether it is due to changes in antennal structure, or *BmMed6* mediated transcriptional changes in OR genes, or a combination of these two factors. The causal relationship requires further investigation, and additional research to confirm. The morphogenesis of male silkworm moth antennae eventually differentiating into a complex plumose structure containing a large number of olfactory receptors. Previous research revealed that *intersex*, as a subunit of the Mediator complex, regulates imaginal disc development in silkworms, with mutations leading to antenna abnormalities.^30^ Our current study found similar antenna deformities in *BmMed6* mutants, emphasizing the critical role of the Mediator complex and suggesting it is a potential new target gene for pest control.

In conclusion, the results of this study provide insights into the functional study of *BmMed6* in silkworms. *BmMed6* not only involved silkworms’ growth and development but also regulated antennal morphology, thereby influencing olfactory perception and mating behavior, which has not been reported previously. It provides important evidence and significance to the future study of the Mediator complex in insects. As a representative model insect within the Lepidoptera, the impact of *BmMed6* on silkworm antennae development suggests it as a potential gene target for pest control strategies, particularly through its influence on mating behaviors.

### Limitations of the study

It is essential to recognize our understanding remains limited. We observed the impact of *BmMed6* on antennal development and mating behavior, but it is unclear whether these changes are due to antennal structure alterations, BmMed6-mediated gene expression changes, or both. Moreover, our transcriptome analysis highlighted differences in cuticle structure, sensory perception of smell, and olfactory receptor activity pathways; these findings require further confirmation. Additionally, we only investigated the impact on antennae and did not investigate the effects on development or body size.

## Resource availability

### Lead contact

Further information and requests for resources and reagents should be directed to and will be fulfilled by the lead contact, Yongping Huang (insectgroup@sjtu.edu.cn).

### Materials availability

This study did not generate new unique reagents.

### Data and code availability

All data reported in this paper will be shared by the lead contact upon request. This paper does not report original code. Any additional information required to reanalyze the data reported in this paper is available from the lead contact upon request.

## Acknowledgments

We thank all members of the Huang lab for technical assistance and helpful discussions. This work was supported by the National Science Foundation of China (32021001, 32100381, 32400378) and financial support from Bühler Group.

## Author contributions

L.Y.Z., D.H.Y., K.L., and Y.P.H. conceived and designed the experiments. L.Y.Z. and D.H.Y. performed experiments. L.H.T. and X.Y.W. assisted in the rearing of insects. L.Y.Z. wrote the manuscript and all the authors participated in the revision of the manuscript and provided comments.

## Declaration of interests

The authors declare no competing interests.

## STAR★Methods

### Key resources table

**Table:** 

REAGENT or RESOURCE	SOURCE	IDENTIFIER
**Critical commercial assays**		

Animal Tissue PCR Kit	Trans	AD201-01
TRIzol reagent	Invitrogen	15596026
SYBR Green Real-time PCR Master Mix	Yeasen	11203ES08

**Software and algorithms**		

sgRNA target sites designed website	website	https://crispr.dbcls.jp/
GraphPad Prism	GraphPad software	https://www.graphpad.com/

**Other**		

NRK-D90 digital cameras	Nikon	D90
scanning electron microscope(SEM)	Zeiss	Zeiss Merlin Compact
RNA-seq raw data	The SRA data: PRJNA1210497	https://www.ncbi.nlm.nih.gov/sra/PRJNA1210497

### Method details

#### Insects rearing

The larvae strain of *B.mori* was Nistari, a non-diapausing silkworm strain. They were reared with mulberry leaf feeding in 25°C standard conditions room.

#### Plasmid construction

We used the CRISPR/Cas9 system to construct the *BmMed6* mutants. Transgenic strain U6-sgRNA (*IE1-DsRed-U6-sgRNA1-U6-sgRNA2*) was used to express two sgRNA. *BmMed6* has five exons, we chose two target sites according to the 5′-G(N_19_)NGG -3′ rule.^57^ The sgRNA target sites were designed using the website (https://crispr.dbcls.jp/). The sgRNA sequences and primers are available in ST1. The transgenic strain nos-Cas9 was constructed previously (IE1-EGFP-nos-Cas9).^58^ We microinjected a mixture of transgenic plasmid, helper plasmids and piggyBac transposon mRNA. Each vector had a concentration of 300 ng/μL. The injection was performed into preblastoderm G0 embryos within 8 h.^59^ The G0 moth produced G1 from sib-mating. We selected the red fluorescent marker-DsRed-positive G1 moth to mate with the nos-Cas9 line. Finally, we acquired the *BmMed6* mutants, which were used in subsequent experiments.

#### Mutagenesis analysis

DNA was extracted from tissue, and a PCR reaction for genomic DNA was performed using the Animal Tissue PCR Kit(Trans, China). Mix extracted genomic DNA with PCR master mix, primers, and nucleotides. Verifying the amplification of the expected DNA fragment through agarose gel electrophoresis. Sequencing the PCR product and comparing it with the reference sequence to identify mutations. Primers were listed in Table S1.

#### Morphologic observation

The phenotypic analysis primarily involved examining the morphology of body size and antenna shape using a light microscope. Photographs were captured using NRK-D90 (B) or DS-Ri1 digital cameras (Nikon, Tokyo, Japan). The antennae of silkworms were dissected, washed with ddH_2_O, and air dried at room temperature. They were observed by scanning electron microscope (Zeiss Merlin Compact, Germany).

#### Sample preparation for RNA-seq analysis

Collecting silkmoth antennae with forceps, washing three times with PBS, and adding them to the TRIzol reagent (Invitrogen, USA). Total RNA was extracted from the antennae of twenty individual adult silkworms from WT and *BmMed6* mutant and mixed together. Total RNA was used for mRNA sequencing. Then it was enriched and subsequently fragmented. This processed RNA was then utilized for cDNA synthesis and the construction of a library. The library was sequenced utilizing Illumina technology, and the raw data obtained underwent qualification, filtering, and mapping to the reference silkworm genome database. Library construction, sequencing and analysis of transcripts were carried out in Sangon Biotech (Shanghai, China).

#### Illumina sequencing

Sequencing uses the Illumina Xten platform (Hiseq 2500). After the sequencing raw data is quality controlled, mapping is mapped to the reference genome sequence. The reads mapping to the genes are counted and the expression level of genes is calculated. The Log2 (FPKM) (fragments per kilobase per million fragments mapped) value was used for analysis. Gene expression differences, differential genes GO, KEGG functional enrichment analysis, KEGG pathway and other analyses were performed. The GO, KEGG were conducted via the website https://david.ncifcrf.gov/home.jsp, and the resulting data was visualized using https://www.bioinformatics.com.cn,^60^ an online platform specialized in data analysis and visualization.

#### RNA isolation and quantitative reverse transcription PCR (qRT-PCR)

Total RNA was extracted from the different tissues of silkmoth using TRIzol reagent. cDNA (TRANSGEN, AE311-03) synthesis and qRT-PCR performed by SYBR Green Real-time PCR Master Mix via (Cat No.11203ES08; Yeasen, Shanghai,China). The primer sequences are presented in Table S1.

#### Experimental setup for moth mating behavioral assays

Silkmoths were housed in a plastic container measuring 10 × 17 × 7 cm. For each assay, The female moth or paper with 10μL of diluted bombykol/bombykal was placed at the center of the container. To evaluate the response, male wild-type and male mutant moths were placed on opposite sides of the container, and a dividing line was in the middle.^43^ The behavior of the moths was observed, and the number of moths that crossed the dividing line was counted, and those that reached the center were considered responsive. The number of responsive moths was recorded, and the response index was calculated. This index was determined as the percentage of responsive moths out of the total number of male moths tested in that particular condition. All assays were replicated a minimum of three times to ensure the reliability of the results.

### Quantification and statistical analysis

Data were analyzed by GraphPad Prism and presented as mean ± SEM. Statistically significant differences were determined by paired Student’s t-test, (∗, *p* < 0.05; ∗∗, *p* < 0.01; ∗∗∗, *p* < 0.001; ∗∗∗∗, *p* < 0.0001).

## Supplemental information

Document S1. Figures S1–S5 and Table S1

Video S1. Video demonstration of mating behavior assay

Video S2. Second example- video demonstration of mating behavior assay
