# Supplementary material for: BmMed6 modulates mating behavior by ORs and antennae structural genes in the silkworm
Source: iScience. 2025 Feb 13;28(4):112017. doi: 10.1016/j.isci.2025.112017 (PMC11978329; doi:10.1016/j.isci.2025.112017)
Supplement: Document S1. Figures S1–S5 and Table S1 [file mmc1.pdf]

iScience, Volume *28*

Supplemental information

BmMed6 modulates mating behavior by ORs and antennae structural genes in the silkworm

Zhang Liying, Yang Dehong, Tang Longhao, Wei Xiangyi, Li Kai, and Huang Yongping

Supporting information

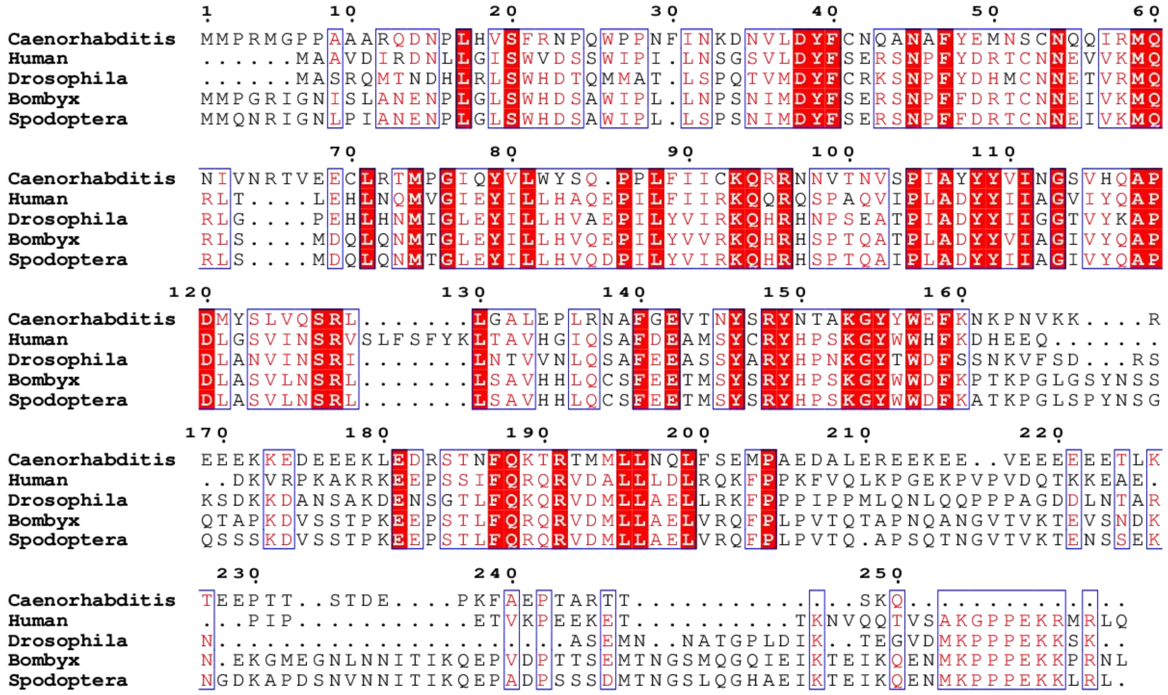

Figure S1. A multiple sequence alignment of Med6 protein sequences from five species was conducted. Conserved residues among all sequences were highlighted with blue boxes.

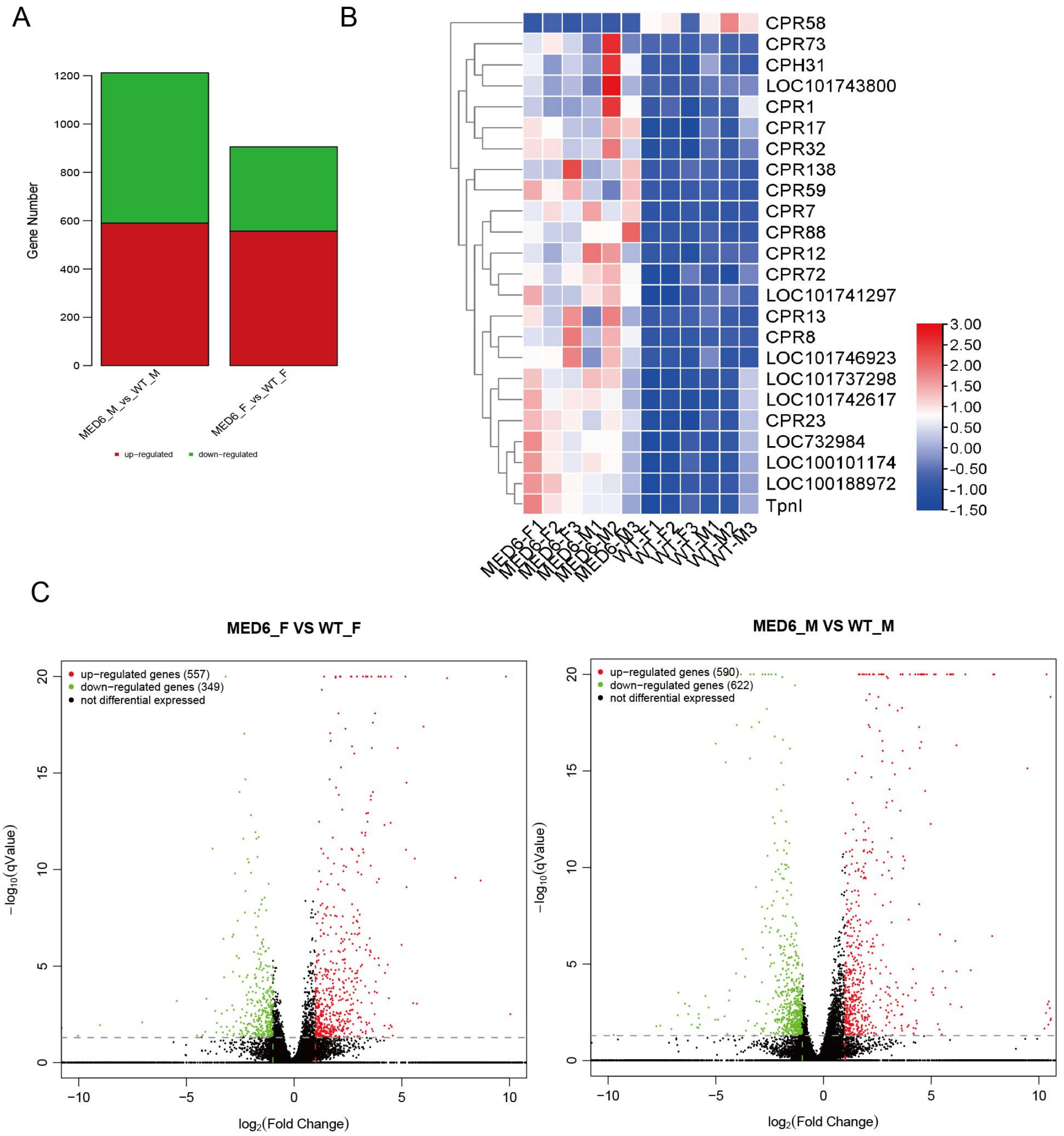

Figure S2. (A) Statistics of differentially expressed gene mRNAs. (B) Heatmap of structural constituent of cuticle. (C) Volcanic maps for differential expressed mRNAs. Red dots, significantly genes. Green dots, significantly downregulated genes. Black dots, nondifferentially expressed genes.

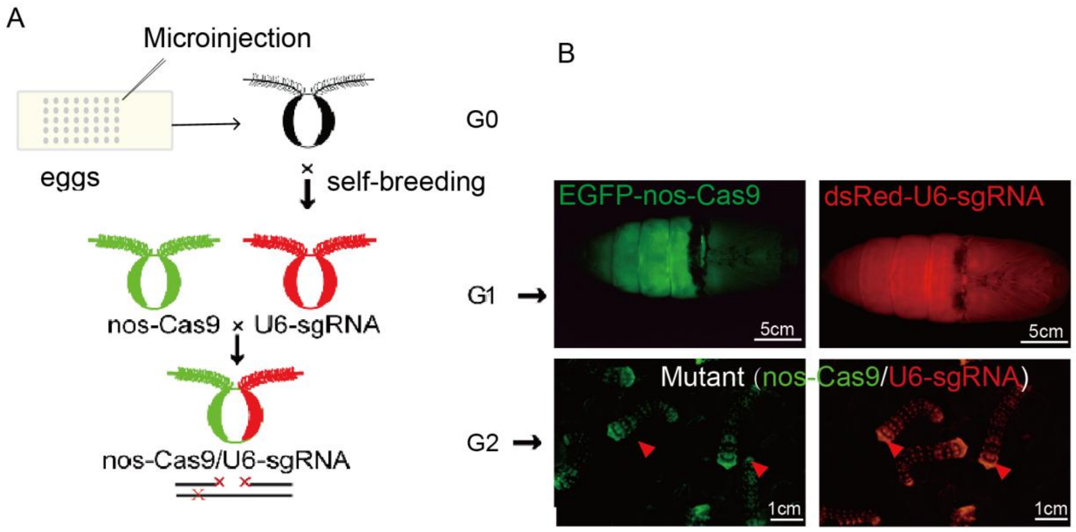

Figure S3. (A) Schematic of the workflow for the identification of mutant. Constructs were injected into silkworm eggs. Fluorescent signals were analyzed using stereomicroscopy. (B) Fluorescent images. The dsRed-positive G1 line was mated with the nos-Cas9 line to generate G2 mutants exhibiting both red and green fluorescence.

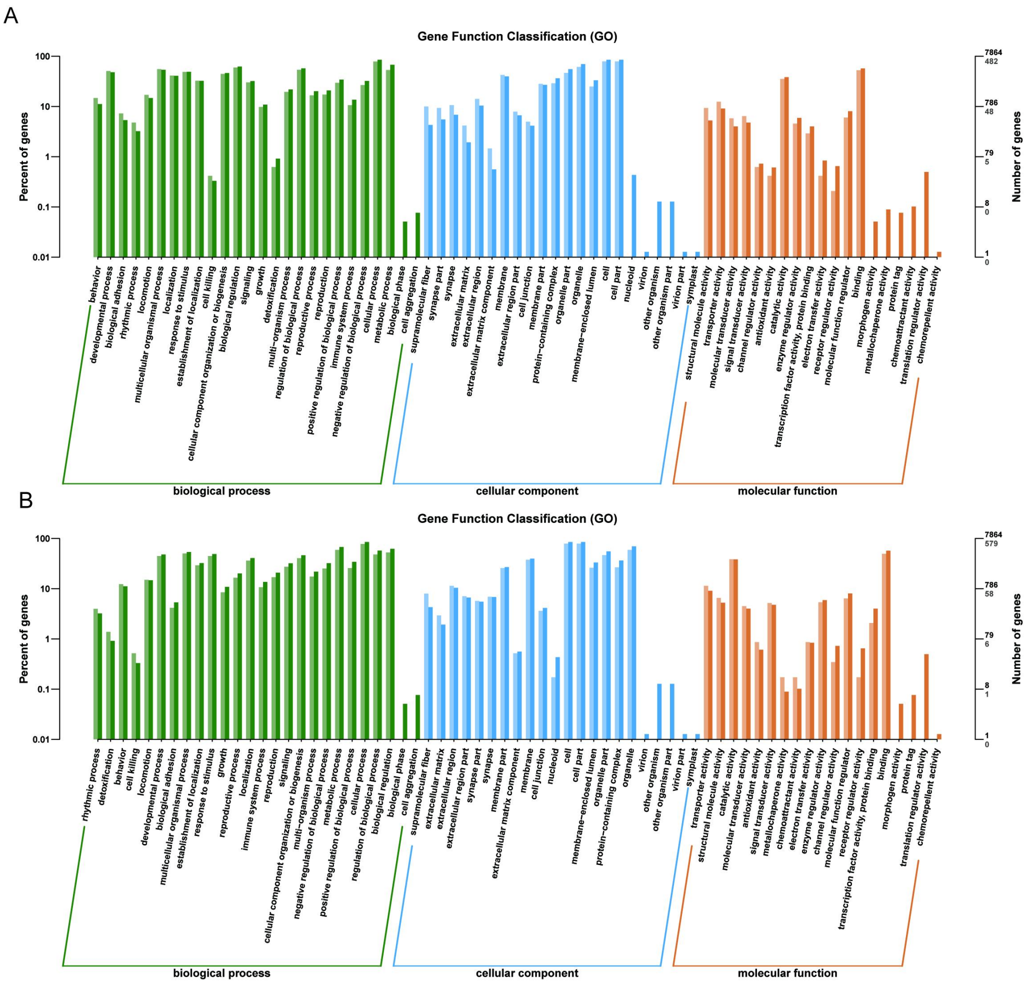

Figure S4. (A) GO analysis of the transcriptome from female antennae. (B) GO analysis of the transcriptome from male antennae.

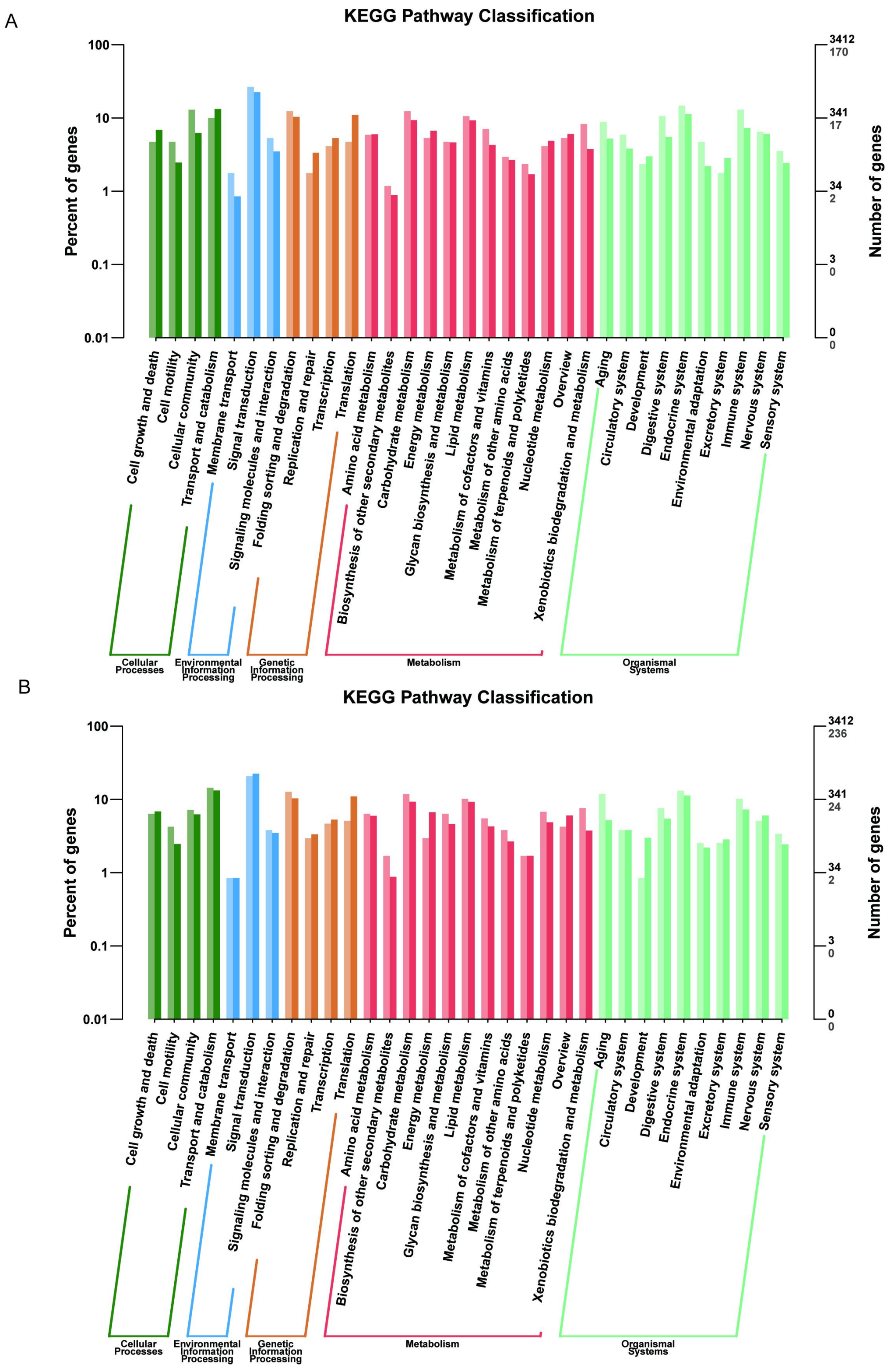

Figure S5. (A) KEGG analysis of the transcriptome from female antennae. (B) KEGG analysis of the transcriptome from male antennae

Table S1. Primer List

Primer name	Sequence (5’-3’)
Med6-KO-F	GCACCAAAATCTATGGATAAAAGCA
Med6-KO-R	GTGGTTTTTACGGATGTTCCG
RP49-F	TCAATCGGATCGCTATGACA
RP49-R	ATGACGGGTCTTCTTGTTGG
Q-MED6-F	GGCATGATTCAGCATGGATACCG
Q-MED6-R	CGCACAACATAGAGAATTGGCTCTT
OR12-F	TGTTAGATTGCGAAGAGTTCAAGC
OR12-R	GACCAATAACCAGCATGAAACA
OR13-F	CACTGTCAGTCCCGGTTGAG
OR13-R	TACCAAACCAGCATGGGACC
OR16-F	TTTACTCCGCGCTGGCTATT
OR16-R	CGTTTGGTCCAAGACAGCAA
OR18-F	CGTTCCTCAGCGTTGCTCTA
OR18-R	TGTCCACGGGCCACAATATC
OR34-F	GGCACGTACCCATACACGAA
OR34-R	ATTTGCGAATAATCGGCGGC
OR39-F	GCTGATTCAGGTCTCGGTGG
OR39-R	GCAACAGTAGCAACACGACC
OR42-F	GAACGCCGTCCTCATTTTCG
OR42-R	CCACGGGATCCAGGAATCAG
OR5-F	AGGATTTTCAGGCTTCGGTAC
OR5-R	GTTATGATAGTGTGGCCGAGG
OR56-F	GGTGGCGCAGTTTAACAAGG
OR56-R	GCGACTGACTGAGCAGTGT
OR59-F	GCACCACCCAGCTTAGGATT
OR59-R	AGCAATTCGTGAGTCACCGT
OR6-F	CAAGGTGCTTGCCCATGTTT
OR6-R	AGCCGGACGACATTACACTG
OR7-F	CAGGCTTCGGTACATCCTGG
